# The Post-Synaptic Density of Human Postmortem Brain Tissues: An Experimental Study Paradigm for Neuropsychiatric Illnesses

**DOI:** 10.1371/journal.pone.0005251

**Published:** 2009-04-16

**Authors:** Chang-Gyu Hahn, Anamika Banerjee, Matthew L. MacDonald, Dan-Sung Cho, Joshua Kamins, Zhiping Nie, Karin E. Borgmann-Winter, Tilo Grosser, Angel Pizarro, Eugene Ciccimaro, Steven E. Arnold, Hoau-Yan Wang, Ian A. Blair

**Affiliations:** 1 Department of Psychiatry, University of Pennsylvania, Philadelphia, Pennsylvania, United States of America; 2 Centers for Cancer Pharmacology and Excellence in Environmental Toxicology, University of Pennsylvania, Philadelphia, Pennsylvania, United States of America; 3 Institute for Translational Medicine and Therapeutics, University of Pennsylvania, Philadelphia, Pennsylvania, United States of America; 4 Department of Physiology & Pharmacology, City University of New York Medical School, New York, New York, United States of America; 5 Children's Hospital of Philadelphia, Philadelphia, Pennsylvania, United States of America; The Research Institute for Children at Children's Hospital New Orleans, United States of America

## Abstract

Recent molecular genetics studies have suggested various trans-synaptic processes for pathophysiologic mechanisms of neuropsychiatric illnesses. Examination of pre- and post-synaptic scaffolds in the brains of patients would greatly aid further investigation, yet such an approach in human postmortem tissue has yet to be tested. We have examined three methods using density gradient based purification of synaptosomes followed by detergent extraction (Method 1) and the pH based differential extraction of synaptic membranes (Methods 2 and 3). All three methods separated fractions from human postmortem brains that were highly enriched in typical PSD proteins, almost to the exclusion of pre-synaptic proteins. We examined these fractions using electron microscopy (EM) and verified the integrity of the synaptic membrane and PSD fractions derived from human postmortem brain tissues. We analyzed protein composition of the PSD fractions using two dimensional liquid chromatography tandem mass spectrometry (2D LC-MS/MS) and observed known PSD proteins by mass spectrometry. Immunoprecipitation and immunoblot studies revealed that expected protein-protein interactions and certain posttranscriptional modulations were maintained in PSD fractions. Our results demonstrate that PSD fractions can be isolated from human postmortem brain tissues with a reasonable degree of integrity. This approach may foster novel postmortem brain research paradigms in which the stoichiometry and protein composition of specific microdomains are examined.

## Introduction

Recent studies in neuropsychiatric illnesses have implicated various trans-synaptic mechanisms [1], [2], [3], [4], [5], [6], [7], for which protein compositions in sub-cellular microdomains are crucial [8], [9], [10], [11], [12]. The postsynaptic density (PSD) is a particularly interesting microdomain since multiple candidate genes for psychoses and mood disorders converge on its signaling mechanisms [5], [6], [7], [13]. Thus, the ability to isolate the PSD, particularly when combined with proteomic methods, will greatly aid our understanding of neuropsychiatric illnesses. Indeed, such strategies have been extensively utilized for the PSD derived from rodent brains [14], [15], [16], [17]. We, and others, have recently applied similar approaches to human postmortem brains [18], [19], but the feasibility and validity have not been fully elucidated. In this study, we evaluate a research paradigm to isolate biochemical fractions enriched in the PSD of human postmortem brains, which can then be used as a platform to profile the stoichiometry of specific receptor complexes, when combined with a proteomics approach.

The PSD is a highly organized biochemical apparatus attached to the postsynaptic membrane, which can be visualized as an electron dense thickening under electron microscopy [20], [21]. The PSD contains glutamatergic receptors, N-methyl-D-aspartic acid (NMDA), alpha-amino-3-hydroxy-5-methyl-4-isoxazolepropionic acid receptor (AMPA), the group I metabotropic glutamate receptors (mGluRs) and numerous signaling molecules [22], [23]. As a molecular scaffold, the PSD brings together various sets of signaling molecules and modulates their interactions and their activities.

The PSD is relatively insoluble in non-ionic detergents and can be purified by differential centrifugation [24], [25]. Various protocols have been used to isolate the PSD from rodent brains based on a fundamental principle of: a) purification of synaptic membranes, b) extraction of the membranes with non-ionic detergents and c) separation of the resultant disk-shaped protein structures. Protocols have often differed in the degree of detergent extraction, thereby determining the inclusiveness of proteins in the final fractions. These include Triton X-100 or N-lauryl sarcosinate and the use of harsher detergents retrieves a more stripped down version of proteins [24], [25]. Indeed, variations of these methods allowed separation of fractions containing synaptic vesicles and synaptic membranes from the PSD [26], [27].

Protein composition and stoichiometry of protein complexes can be profiled using proteomic analysis. Several groups have analyzed the rodent PSD using 2D fluorescence difference gel electrophoresis (2D DIGE) or LC-MS/MS and identified sets of proteins, including known PSD proteins previously characterized by the immunoblot method [28], [29], [30], [31]. However, depending on the methods of fractionation and proteomics analysis, these studies have reported variable numbers of proteins ranging from a few hundred to more than a thousand. Posttranslational modifications (PTM) appear to be maintained through the rigorous biochemical fractionation procedure, and groups have identified phosphorylation sites on PSD proteins [15], [17], [31], [32]. These studies suggest that heavy isotope labeled synthetic peptides could permit absolute quantification (AQUA) of peptides and posttranslational modifications of proteins in this microdomain.

Biochemical fractionation of postmortem brain tissues faces special challenges due to various confounding factors inherent to these tissues [5], [33]. They include prolonged agonal state, complicating medical conditions, the post-mortem interval (PMI), inconsistent pH, and duration of storage and temperature of the tissues. These conditions are thought to disrupt the integrity of proteins, ultrastructures or microdomains [34], [35], [36]. Therefore, few attempts have been made to isolate the PSD or other sub-cellular fractions from human postmortem brains [37]. In the present study, we have isolated PSD fractions from human postmortem brain tissues and verified the enrichment, protein composition, and integrity of ultrastructures using Western blot, electron microscopy and proteomic analysis. We propose this approach as a new paradigm for postmortem brain study permitting analysis of stoichiometry and protein-protein interactions in this microdomain.

## Results

### Protein enrichment in PSD fractions

**Figure 1:**
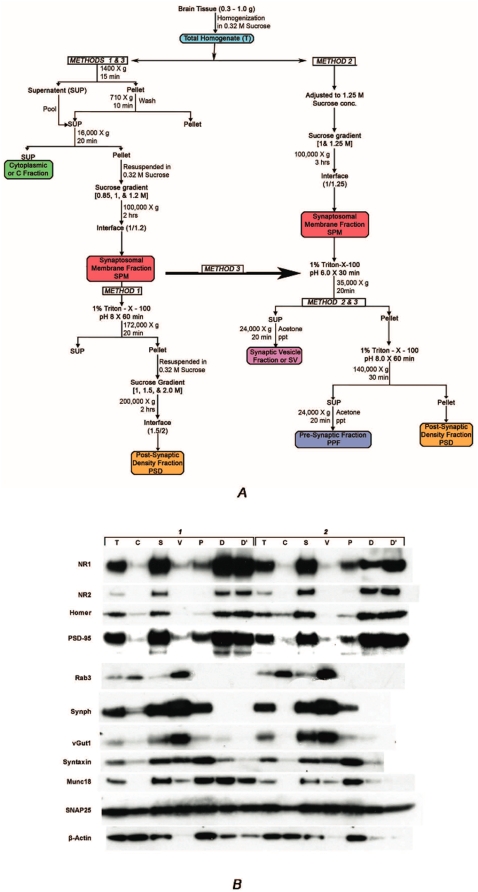
The postsynaptic density (PSD) can be successfully isolated from human postmortem brains. (A) Diagram of fractionation procedures. (B) Immunoblotting of fractions. Postmortem PFC tissues from two human subjects were fractionated by the procedure employing sucrose density gradient and detergent extraction (Method 1) and by the procedure using pH based differential extraction of synaptic membranes (Method 3). Various fractions from Method 3 and the PSD fraction from Method 1 were analyzed by immunoblotting. *Abbrevations: T: total tissue extracts, C: cytosolic extracts, S: SPM, synaptosomal membrane, V: SV, vesicular fraction, P: PPF: presynaptic fraction, D′: PSD isolated by the method 1, ’: D:PSD isolated by the method 3. NR: NMDA receptor, Synph: synaptophysin, vGlut1: vesicular glutamate reuptake site.*

Postmortem human PFC tissues were fractionated following the protocols of Carlin et al [25] and Phillips et al [14], [26], as well as by combining these two methods with some modifications (named Method 1, Method 2 and Method 3 respectively). All three protocols first separated synaptic membrane fractions (SPM) using a sucrose density gradient. In Method 1, the SPM was extracted with Triton X-100 and then fractionated to the PSD with a second sucrose density gradient (Figure 1A). In Methods 2 and 3, the SPM was further fractionated by Triton X-100 extraction first at pH 6, then at pH 8 (Figure 1A). The soluble fractions obtained after the pH 6 and pH 8 Triton X-100 extractions were designated the synaptic vesicular fraction (SV) and the presynaptic membrane fraction (PPF), respectively. Insoluble fractions remaining after the Triton X-100 extraction were defined as the PSD fraction.

All three methods produced fractions highly enriched in the PSD from human postmortem brains. Figure 1B shows that PSD proteins, NMDAR1, NMDAR2, Homer and PSD-95, were enriched in the PSD fractions isolated by Method 1 (D′) or by Method 3 (D), while these proteins are much less represented in the fractions SV (V) or PPF (P). In contrast, presynaptic proteins, Rab3, NRG1, synaptophysin, vGAT, vGlut1, were mostly enriched in the SV fraction but not in the PSD fractions (D′ or D).

**Figure 2:**
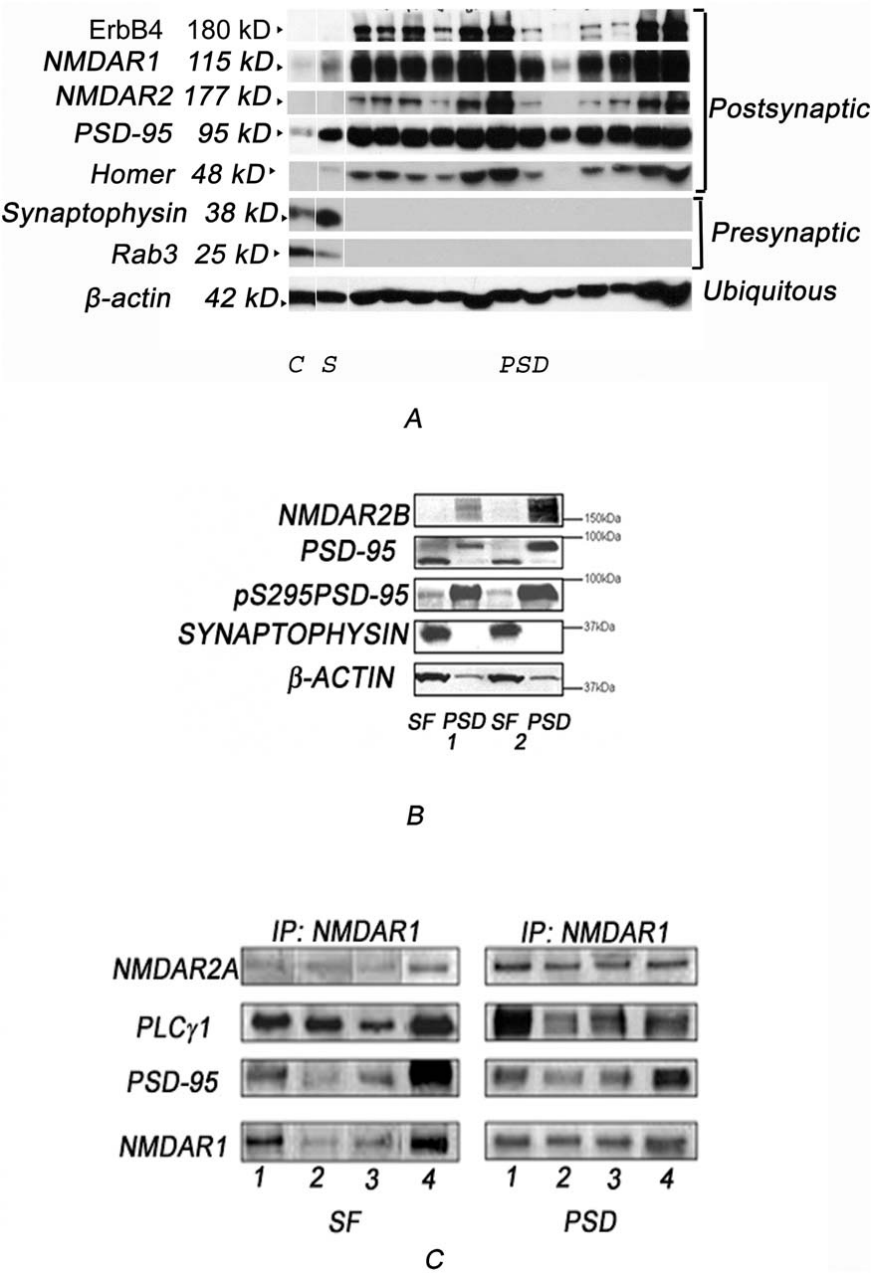
PSD protein composition and protein-protein interactions remain stable through fractionation. (A) PFC tissues from 12 subjects were processed using Method 3. PSD fractions were immunoblotted for postsynaptic and presynaptic molecules. (B) A representative immunoblot for SF and PSD fractions from 2 subjects, fractionated by Method 2, that were probed for PSD proteins, PSD-95 and NR2B, and a presynaptic protein, synaptophysin. β-actin was used as loading control. (C) SF and PSD fractions (Method 2) obtained from 4 subjects without neuropsychiatric illnesses were immunoprecipitated for NMDAR1 and probed for NMDR2A and PLCγ1 PSD-95, NMDAR1.

To assess enrichment of the PSD, PSD fractions extracted by Method 2 were analyzed compared to post-nuclear lysates (referred to as synaptosomal fractions, SF) for the relative quantities of PSD proteins compared to β-actin. Ratios of PSD-95, NR1 and NR2B, with respect to β-actin levels, were approximately 30 fold higher in PSD fractions than those in SF (Fig 2B).

Three methods, as described in Fig 1A, were compared for the yield and purity of fractions. From 500 mg of brain tissues, Method 1 yielded 0.3 to 0.5 mg of SPM and 0.03–0.06 mg PSD, Method 2 produced 1.4 mg SPM and 0.4 mg PSD and Method 3 yielded approximately 0.8 mg of SPM, 0.5 mg of SV, 0.25 mg of PPF and 0.25 mg of PSD fractions. The purity of PSD fractions was determined by the extent to which they contained presynaptic protein and synaptic membrane bilayers. Ratios of synaptophysin with respect to PSD-95 were compared between methods using immunoblot analysis of fractions. The ratio of synaptophysin to PSD-95 was lower in Method 1 than in Methods 2 or 3, indicating that the purity of the fraction was superior in Method 1 by this measure. As another measure of purity we quantified fragments of synaptic membrane bilayers under EM. In eight randomly selected EM fields (magnification 20,000×), 20% fewer synaptic membrane bilayers were observed in Method 1 than in Method 2. Together, these indicate that the fraction purity was superior in Method 1. PSD yield was unaffected by PMI, age or freezing time of the brain tissues (Figure S1).

### Stability of protein interactions and PTM

Postmortem confounds differ among subjects and can lead to individual variability in the integrity of sub-cellular fractions derived from the tissues. To test this possibility, we isolated PSD fractions from the postmortem PFC of 12 subjects and compared presynaptic and postsynaptic proteins. Age of the subjects varied between 67 and 92 (79±2.8). PMIs ranged from 3.5 to 22 (9.6±2.3) (Table 2), the −80°C storage time (after death) was from 5 to 17 years (10.2±1.9). Figure 2A shows that the pre-synaptic molecules rab3 and synpatophysin were almost undetectable in all PSD fractions, while PSD proteins, NR1, NR2A, erbB4, homer and PSD-95 were highly enriched. The ratios of the intensity of PSD-95 bands with respect to β-actin were calculated as a measure of enrichment of PSD proteins. In PSD samples obtained from 12 subjects, the ratios of PSD-95/β-actin was 1.24±0.08, of NR1/β-actin was 1.72±0.74 and of homer/β-actin was 0.72±0.07. Overall, the standard errors of these ratios were less than 15% of the mean for each molecule.

Isolated PSD fractions can provide valuable information on protein-protein interactions in the microdomain. To test whether such molecular interactions are maintained during the rigorous biochemical fractionation, we conducted an immunoprecipitation (IP) experiment with PSD fractions obtained from 12 subjects (Figure 2C). Those fractions were immunoprecipitated with antibodies for NMDA receptor (NR1) and were probed for PSD-95, phospholipase C (PLCγ) and NR2A. Ratios of the intensities of the bands for PSD-95 with respect to those for NR1 were 1.6±0.18 in SF fractions, and 1.0±0.09 for PSD fractions (data not shown). Ratios of the intensities of the bands for PLCγ with respect to those for NR1 were 5.2±0.54 in SF fractions and 1.0±0.11 for PSD fractions (data not shown). The associations of the proteins differ between SF and PSD fractions, yet the ratios of SEM over means were overall comparable between the fractions, suggesting that biologically significant interactions are still maintained in the PSD fractions. Protein-protein interactions in the PSD were unaffected by PMI age or freezing time of the tissue samples (Figure S2).

### EM analysis of fractions

**Figure 3:**
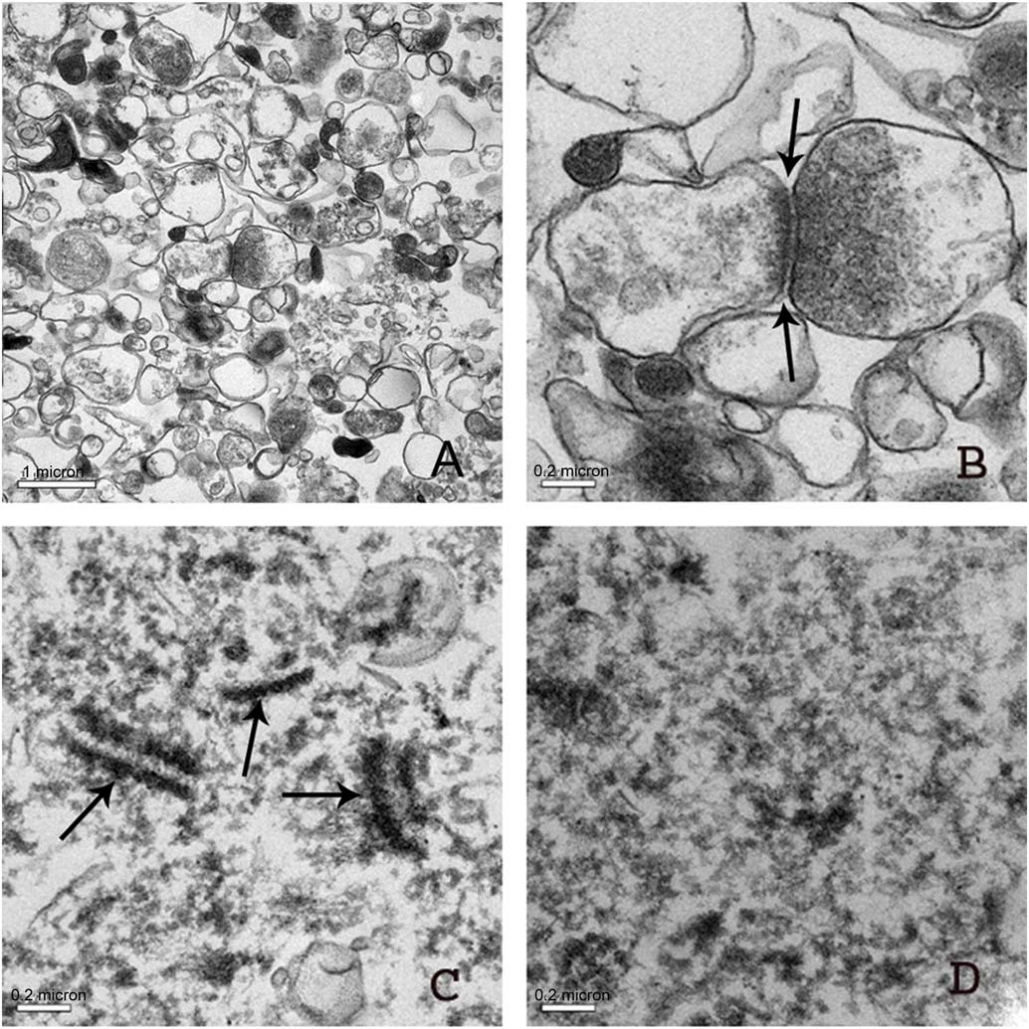
Electron micrographs of osmicated insoluble pellets obtained by Method 3). (A, B) Thin sections of SPM pellets show surprisingly intact synaptosomes and intact synaptic vesicles (A, B) as well as filamentous crossbridges (see arrows in B). (C) Synaptic membrane fractions extracted by Triton-X 100 at pH 6.0 show paired electron dense profiles representing synaptic junctions (see arrows in C). (D) PSD fraction obtained as an insoluble phase after synaptic membranes were extracted with Triton-X 100 at pH 8.0. Note that presynaptic specialization is removed and that the PSD is thinner than shown in A and B.

The integrity of isolated fractions was tested using EM. Figure 3A and B show a thin section electron micrograph of SPM and demonstrate strikingly intact synaptsomes that contain synaptic vesicles and filamentous connections to other synaptosomes. Figure 3C represents the soluble fraction of pH 6 Triton X-100 extraction and shows paired pieces of electron dense membranes. The more electron dense membrane is thought to be postsynaptic. Figure 3D shows that the PSD fraction is largely devoid of the fine substructures of synaptic junctions.

### LC-MS/MS Data

Two biological samples of PSD fractions, enriched by Method 2, were analyzed by 2D LC-MS/MS. Peptide identifications were assigned by SEQUEST (ThermoFisher) and peptide and protein probabilities determined by Empirical Bayes Protein Identifier (EBP) [38]. At a 1% false positive threshold 1863 nonredundant proteins or protein groups were identified. A separate analysis utilizing a reverse database yielded near identical protein identifications at a 1% false positive cut off (data not shown).

Inspection of the identifications revealed proteins with well-theorized functions in the PSD, as identified by review of recent literature [22], [29], such as the Discs Large Homologs (IPI00552511, IPI00647950 & IPI00790650), Homer (IPI00003566), SHANK (IPI00019794), G proteins (e.g. IPI00290928), glutamate receptors (e.g. IPI00297933), and various kinases (e.g. IPI00007128) and phosphatases (e.g. IPI00008380). Peptides from 36 consensus PSD proteins, as identified in Collins et al. [39], were selected and confirmed by spectra inspection and/or targeted MS2 analysis of peptide ions (Table 1). It is of note that many proteins unlikely to be present in the PSD were evidenced, including pre-synaptic, mitochondrial and translational proteins, indicative of the limitation in selectivity of the applied separation method. The complete data set can be viewed at Peptide Atlas, sample IDs 040804_HumanBrain and 052104_HumanBrain. For a complete list of proteins and protein groups see: http://spreadsheets.google.com/pub?key=pDgU17zRNrsAy-GrFicBkLQ

**Table 1:** Peptides for consensus PSD proteins were observed in the PSD fractions of human postmortem brains.

		%	Protein	IPI
	Description	Cov	Probability	Accession
1	A-kinase anchor protein 5	18	1.00	IPI00307794
2	Brain Creatine Kinase	72	1.00	IPI00022977
3	Calmodulin	47	1.00	IPI00075248
4	CAMKII Alpha	43	1.00	IPI00877169
5	CAMKII Beta	40	1.00	IPI00334271
6	DLG1/SAP97/GKAP	7	1.00	IPI00552511
7	DLG2/PSD-93/Chapsyn-110	13	1.00	IPI00647950
8	DLG4/PSD-95	24	1.00	IPI00790650
9	Dynamin	59	1.00	IPI00887273
10	Glutamate receptor, ionotropic, AMPA 2	11	1.00	IPI00219216
11	Glutamate receptor, ionotropic, AMPA 3	9	1.00	IPI00298700
12	Glutamate receptor, ionotropic, NMDA 2A	3	1.00	IPI00029768
13	Glutamate receptor, ionotropic, NMDA 2B	6	1.00	IPI00297933
14	Glutamate receptor, metabotropic, 5	5	1.00	IPI00296244
15	Gαi o	40	1.00	IPI00398700
16	Gαi 2	27	0.97	IPI00748145
17	Gβ 1	39	1.00	IPI00026268
18	Gβ 2	47	1.00	IPI00003348
19	Gβ 5	19	1.00	IPI00745232
20	Heat Shock Protein 70 12A	50	1.00	IPI00011932
21	Homer 1	21	1.00	IPI00003566
22	Inositol 1,4,5 Triphosphate receptor	5	1.00	IPI00333753
23	L-type calcium channel	10	1.00	IPI00219983
24	PKCα	28	1.00	IPI00385449
25	PKCγ	27	1.00	IPI00007128
26	PKCε	20	1.00	IPI00024539
27	Protein phosphatase 1	23	1.00	IPI00045550
28	Protein phosphatase 2	20	1.00	IPI00000030
29	Protein tyrosine phosphatase, receptor type D	8	1.00	IPI00375547
30	Proto-oncogene tyrosine-protein kinase/Src	10	1.00	IPI00641230
31	Ras-related protein Rab-3A	12	1.00	IPI00023504
32	SH3 and multiple ankyrin repeat domains protein 3/SHANK3	14	1.00	IPI00847618
33	Rap guanine nucleotide exchange factor 2/Rap GEF2	12	1.00	IPI00853219
34	Ras GTPase-activating protein/SynGap	17	1.00	IPI00177884
35	R-type calcium channel	4	1.00	IPI00218338
36	Voltage-dependent anion-selective channel	21	1.00	IPI00216024

Peptide identifications were confirmed by inspection of MS2 spectra and targeted MS2 analysis of peptide parent masses. Description: IPI identifier. % Cov: Percent of protein sequence covered by observed peptides. Peptides reported here have also been observed in at least three other analyses of mammalian PSD preparations as described in Colins et al [39].

**Table 2:** Subject Information.

RX	Sex	Age	Race	PMI	Medication
N	F	74	W	3.5	no data
N	M	67	W	8	no data
N	F	74	W	6	no data
N	M	73	B	8	no data
N	M	86	W	7	no data
N	F	85	W	11	no data
N	F	80	W	15	no data
N	M	70	W	19	no data
N	F	89	B	7	no data
N	F	91	W	11.5	no data
N	F	72	W	7	Valcade
N	M	86	W	7	no data
N	F	83	B	22	no data
N	F	67	W	5.5	no data
N	F	92	W	5	no data
N	M	75	W	17	no data
S	F	75	W	9	Haloperidol
S	M	81	W	5	no medication
S	F	82	W	12	Clozapine
S	F	88	C	7.5	no medication
S	M	69	W	13.5	Prozac, Trazodone
S	F	76	W	9	no data

N: Normal, S: Schizophrenia, F: Female, M: Male, B: Black, W: White, C: Caucasian

## Discussion

The PSD contains molecular machineries for NMDA, AMPA and mGlu receptors, all of which have been implicated in the pathophysiology of various neuropsychiatric illnesses [40], [41], [42], [43]. Thus, examining the PSD of patients' brains will greatly aid pathophysiologic investigations, yet such study paradigms have not been developed for human postmortem brains.

The purpose of this study was to assess whether the PSD can be isolated as a biochemical fraction from postmortem brains and to test the integrity of these fractions. Our results show that methods employing density gradient based purification (Method 1) [14], [26] and pH based differential extraction (Method 2, 3)[25], can produce fractions that are highly enriched in the PSD proteins with a reasonable degree of purity and in a reproducible fashion.

All three methods were effective in producing PSD fractions that are mostly devoid of presynaptic proteins. A few differences between these methods, however, may determine the utility of each depending on the purpose of projects. Methods 2 and 3 are superior to Method 1 in the yield of PSD fractions and therefore are more appropriate when the availability of the tissues is limited, while Method 1 is recommended when the issue of purity is critical.

EM examination of subcellular fractions showed relatively intact ultrastructures in subcellular fractions from postmortem brains. In SPM fractions, synaptosomes were well formed, containing intact synaptic vesicles. In the PPF and much less frequently in the PSD, scaffolds of opposing presynaptic and postsynaptic membranes were observed, as shown in rodent counterparts [14], [24], [25]. In addition, ultrastructural characteristics of PSD fractions appear comparable to those previously reported in rodents [14], [24], [25]. These data support the notion that the PSD, as a microdomain, is relatively well-maintained in postmortem brains, and can be isolated to a reasonable degree of purity. Various confounding factors associated with postmortem brain tissue may affect subcellular ultrastructures and the integrity of proteins [33], [34], [35]. These factors could contribute to variability in the yield and protein composition of PSD fractions, which would make comparisons between individuals or groups unreliable. A few parameters that we have tested in the present study, however, suggest that there is a reasonable degree of consistency in PSD samples of human subjects. First, the rate of enrichment of PSD proteins in the PSD fractions, tested in 12 subjects, appears to be relatively consistent between samples (Figure 2a), while the PMI of the subjects ranged between 3.5 and 22 h and the freezer storage time varied from 5 to 17 years. Second, protein composition of the PSD fractions, as assessed by the ratios of PSD-95 relative to NR1 for example, was grossly similar in the PSD fractions among all subjects. The rate of enrichment and protein composition are relatively consistent, most likely because the PSD is a resilient microdomain that can endure harshness of the fractionation procedures.

PSD fractions of postmortem brain tissue may permit the study of protein-protein interactions with some confidence. The results of the NR1 IP showed that the association of NR1 with PSD-95, PLCγ or NR2A in the PSD was almost as consistent among individuals as those in the post-nuclear fractions (Figure 2C as an example). This may suggest that the fractionation procedure of the PSD does not disturb protein-protein interactions. It is of note, however, that the association of NR1 with NR2A and PLCγ was lower in the PSD than in post-nuclear fractions (Figure 2C). This could be due to different protein associations between the two fractions. Alternatively, it may be the case that only more robust protein-protein associations survive during the fractionation procedure of the PSD, although the selective process is still consistent among samples.

2D LC-MS/MS was employed to further characterize the protein composition of PSD fractions. PSD enrichments from two biological samples were combined in a statistical analysis, which integrates data from consensual and non-consensual peptide identifications in a Bayesian fashion (48). Our list of identified proteins appears highly inclusive, yet provides an extensive coverage of known PSD proteins, as evidenced by the proteins cataloged in Table 1. Combined with Western blot results confirming enrichment of PSD proteins, our 2D LC-MSMS data provide further evidence that PSD proteins can be enriched by a biochemical fractionation of postmortem brain tissue. The number of proteins in the PSD is presently unknown [22]. Using immuno-EM, EM tomography, and scanning transmission EM, Chen et al (2005) and Petersen et al (2003) have estimated that an average PSD with a 360 nm diameter and a total molecular mass of 1.10±0.36 gigadaltons, could be composed of 10,000 proteins of 100kD [44], [45].

Proteomic analyses are prone to detecting contaminants that are either highly abundant in target tissues or co-enriched during biochemical fractionation [22]. While our list of identified proteins is highly inclusive, it may serve as a comprehensive list of proteins in the PSD of human postmortem brains. The next step will be to determine which of these proteins are specific to the PSD or highly enriched in this microdomain. Once confirmed, PSD proteins on the list can be quantitatively evaluated in conjunction with immunoprecipitation and heavy label internal standards.

The ultimate goal of this study was to test a study paradigm in which to examine the PSD of human postmortem brains with respect to its integrity, protein composition, and protein associations. Our results showed surprisingly well-maintained ultrastructures in biochemical fractions, relatively consistent yield of PSD fractions and the stability of protein compositions during the fractionation procedure. Considering various postmortem confounding variables and their effects on a wide array of protein properties, it will still be important to be selective for parameters that are stable enough for comparisons. When such cautions are exercised, however, this approach can provide insights into protein-protein interactions that are critical for glutamatergic and other signaling mechanisms in post-synaptic neurons.

## Materials and Methods

### Subcellular fractionation of postmortem brain tissues

#### PSD fractionation Method 1

One gram of frozen human postmortem brain tissue was homogenized in 5 ml of solution A and the samples were pelleted at 2638 rpm (1400×g) for 15 minutes in Eppendorf 5810R centrifuge. The pellet was re-homogenized in 2 ml of solution A and centrifuged at 1878 rpm (710×g) for 10 minutes. The supernatant was combined with supernatant from the previous step, and centrifuged at 13,800 rpm (16000×g) in Eppendorf centrifuge 5415 C for 20 min. The pellet was resuspended in 750 µl of solution B and overlaid on a three-layer sucrose gradient that consisted of 3 mls each of 1.2 M, 1 M, and 0.85 M sucrose. The gradient was spun at 28,000 rpm (100,000×g) for 2 hrs in a SW 40 Ti rotor using a Beckmann L7 Ultracentrifuge. The band between 1 and 1.2 M interface was collected as SPM. 500 µl of the SPM was diluted 5× with 0.1 mM CaCl2 and centrifuged at 12,000 rpm (15000×g) in Beckmann type 50 Ti rotor for 20 minutes. The pellet was dissolved in 20 mM Tris pH 7.4, sonicated with three 10 second pulses and solubilized in triple detergent as described above. The rest of the SPM (∼1.5 mls) was diluted with 0.1 mM CaCl2 to a total volume of 7.5 mls, followed by the addition of 7.5 mls of 40 mM Tris pH 8.0 with 2% Triton-X 100 to make a final volume of 15 mls (20 mM Tris and 1% Triton-X) and left in cold room with end over end shaking for 60 minutes. The samples were pelleted down at 40,000 rpm (172,000×g) in 50 Ti rotor and resuspended in 1 ml of solution A. This resuspended pellet was overlaid on a three-layer sucrose gradient made up of 3 mls each of 2, 1.5 and 1 M sucrose and centrifuged at 44,000 rpm (200,000×g) in 50 Ti for 2 hrs. The band between 2 and 1.5 M sucrose was collected as PSD. PSD fractions were solubilized in triple detergent with sonication as described above. Protein concentrations in SPM and PSD were determined by Bradford's method.

#### PSD fractionation Method 2

300–350 mg of frozen postmortem brain tissue was dissected and homogenized in 1.5 ml of solution A (0.1 mM CaCl2, 1 mM MgCl2 and 0.32 M sucrose) supplemented with protease and phosphatase inhibitor cocktails (Sigma). This homogenate was adjusted to 1.25 M Sucrose and 0.1 mM CaCl2 to a total volume of 5 ml. 5 ml of 1 M sucrose was overlaid on this and ultracentrifuged at 28000 rpm (100,000×g) for 3 hrs in a SW 40 Ti rotor using a Beckmann L7 Ultracentrifuge. The band at the interface of 1.25 and 1 was collected with a needle as SPM. The SPM was diluted with 5× 0.1 mM CaCl2 and centrifuged at 12000 rpm (15000×g) for 20 minutes. The pellet was solubilzed in 20 mM Tris pH 7.4 supplemented with protease and phosphatase inhibitors, and sonicated with three 10 second pulses. Samples were added with a combination of three detergents, 0.5% digitonin, 0.2% sodium cholate and 0.5% NP-40 (final concentration), which would be henceforth referred to as triple detergent, and the samples were left for end over end shaking for 60 min in 4°C.

For PSD preparation, a 500 µl aliquot of SPM was diluted with ice cold 0.1 mM CaCl2 to 2.5 ml, followed by the addition of 2.5 ml of 40 mM Tris-HCl pH 6 supplemented with 2% Triton-X 100 and protease and phosphatase inhibitors, bringing the final volume to 5 ml with 20 mM Tris and 1% Triton-X 100. The samples were left in cold room rocker for 30 minutes and centrifuged at 18000 rpm (35,000×g) for 20 minutes. The supernatant, designated as the vesicular fraction (SV), was then diluted with 5× chilled acetone and left in −20°C overnight. The pellet was air dried and dissolved in 1 ml CaCl2, then 1 ml 40 mM Tris pH 8 with 2% Triton-X 100 , was added for the final concentration of 20 mM Tris and 1% Triton-X. The sample was left on a rocker for 60 minutes and centrifuged at 36000 rpm (140,000×g) for 30 minutes. The supernatant, designated as the presynaptic membrane fraction (PPF) was acetone precipitated at −20°C overnight. The pelleted PSD was air dried and dissolved in 20 mM Tris pH 7.4. Acetone precipitated SV and PPF fractions were centrifuged at 15000 rpm (24,000×g) for 30 minutes and pellets were dissolved in 500 µl of 20 mM Tris pH 7.4 with triple detergent.

#### PSD fractionation Method 3

One gm of postmortem brain tissue was homogenized in 5 ml of solution A (0.32 M Sucrose, 1 mM NaHCO3, 1 mM MgCl2, 0.5 mM CaCl2 supplemented with protease and phosphatase inhibitor mixtures), which was labeled total homogenate (T). The sample was centrifuged at 2638 rpm (1400×g) for 15 min at 4°C on Eppendorf 5810R, and the supernatant was saved. The pellet was rehomogenized in 2 ml of solution A, centrifuged at 1878 rpm (710×g) for 10 min, and pooled with supernatants from the previous step. Pooled samples were centrifuged at 13,800 rpm (16000×g) in Eppendorf Centrifuge 5415C, and the supernatant was saved as the cytosolic fraction (C). The pellet was resuspended in 750 ml of solution B (0.32 M sucrose, 1 mM NaHCO3 supplemented with protease inhibitor mix and phosphotase inhibitors) and overlaid on a three layer sucrose gradient that consisted of 3 mls of each 1.2 M, 1 M and 0.85 M sucrose. The gradient was spun for 2 hrs at 28,000 rpm (100,000×g) in a SW40Ti rotor in a L7 Beckmann ultracentrifuge. The band between 1.0 and 1.2 M sucrose containing synaptosomes was removed, diluted with 5× solution B and centrifuged for 30 minutes at 18000 rpm (35,000×g) in Beckmann type 50Ti rotor. The interface at 1 M and 1.25 M sucrose gradients was processed as described for the interface between 1.2 and 1.0 M sucrose gradients. Protein concentrations for SPM, SV, PPF and PSD fractions were determined by Bradford's method [46].

### Synaptosomal fractions (SF)

100 mg of brain tissues was homogenized in 5× volume of immunoprecipitation buffer (25 mM Tris HCl pH 7.4, 200 mM NaCl, 2 mM EDTA, 0.5 mM EGTA and cocktails of protease and phosphatase inhibitors, (Sigma)). The samples were centrifuged at 3500 rpm (800×g) in Eppendorf 5415C for 15 min at 4°C. The supernatant was sonicated for 10 seconds on ice and triple detergents were added. The samples were incubated at 4°C for 60 min on an end over end shaker and were spun at 13800 rpm (11000×g) for 10 minutes. The supernatant was diluted with 0.75 mls of IP buffer and saved as synaptosomal fraction (SF).

### Electron Microscopy

Pellets were fixed in 2.5% glutaraldehyde/2% paraformaldehyde, embedded in epon, and post-stained with urayl acetate and bismuth subnitrite. Ultrastructural examination was performed with a JEOL JEM −1010 electron microscope. Images were captured with a Hamamatsu CCD ORCA digital camera, using AMT Advantage software version 5.4.2.308 (Advanced Microscopy Techniques).

### Western blotting and Immunoprecipitation

To test enrichment of PSD proteins by Method 2, the T, C, SPM (S), SV (V), PPF (P) and PSD (D), (also D′: the PSD fractionated by Method 1) fraction protein extracts were separated on 4–12% NuPage precast gel from Invitrogen (Figure 1B).

SF and PSD (fractionated by method 3) of the same human subjects were compared by loading 30 µgs of SF and 5 µgs of PSD protein extract in 7.5% SDS precast gels from Biorad (Figure 2B).

The samples were transferred on PVDF membrane (Millipore) and probed with the following antibodies: mouse monoclonal PSD-95, 1∶1000 (Upstate or NeuroMab, UC Davis, CA), rabbit polyclonal pS295-PSD-95 1∶250 (Abcam), mouse monoclonal synaptophysin 1∶5000 (Chemicon), mouse monoclonal β-actin 1∶5000 (Sigma), rabbit polyclonal ErbB4, goat polyclonal NR1, goat polyclonal NR2A (Santa Cruz) , NR2B 1∶1000 (a gift from Dr. Barry Wolfe, Georgetown University Medical Center, Washington, DC 20057, USA), rabbit polyclonal vGlut1 (Synaptic System), mouse monoclonal Munc18 and RAB3 (BD Biosciences). The blots were developed using chemiluminescent reagents ECL or ECL-Plus, or a combination of both (GE Healthcare). 400 µgs of SF or 10 µg of PSD from the same subject were immunoprecipitated after 2 hours preclearing with Protein A agarose with 6 µg of goat polyclonal NR1 antibody (Santa Cruz) in immunoprecipitation buffer (25 mM Tris-HCl, pH 7.4, 200 mM NaCl, 2 mM EDTA, 0.5 mM EGTA supplemented with protease and phosphatase inhibitors from Sigma) at 4°C overnight. The samples were incubated with 25 µl of Protein A agarose Plus (Pierce) for 2 hrs at RT. The immunoprecipitated samples were separated on a 7.5% SDS precast gel (Biorad) and probed with mouse monoclonal PSD-95, 1∶1000 (NeuroMab), Goat Polyclonal NR1 and NR2A 1∶500 and mouse monoclonal PLCγ1 1∶250 (Santa Cruz).

### 2D LC-MS/MS Analysis of Human PSD Enrichments

Tissue samples were dissected from the prefrontal cortex (Brodmann area 9) of two healthy control human brain samples in order to provide sufficient confidence in protein identifications. The PSD was enriched by Method 2, for increased yield and inclusiveness, and purity confirmed by western blot analysis of PDZ domain and synaptophysin (Figure 1B). Each pellet was washed 3 times with 20 mM Tris pH 7.4 at 4°C and resuspended in 25 mM ammonium bicarbonate with 6 M guanidine hydrochloride. Protein concentration was determined by Coomassie Plus reagent (Promega, Madison, WI) with a BSA standard curve read at 595 nm on an EL808 Microplate Absorbance Reader (BioTek, Winooski, VT. Two enrichments per biological sample were pooled to give 600 µg PSD protein. 2D LC-MS/MS was performed using a variation of the method described by Trinidad et al. [15]. Cysteine side chains were reduced and alkylated by incubation with 5 mM dithiothreitol (DTT) for 45 min at 60°C and 15 mM iodoacetamide for 45 min at room temperature in the dark. The mixture was diluted to a final concentration of 1 M guanidine with 25 mM ammonium bicarbonate and 75 µg modified trypsin (Promega, Madison, WI). The mixture was adjusted to a pH of 8.0 and incubated for 12 hrs at 37°C. Digests were desalted with a Peptide Macrotrap Cartridge (MICHROM Bioresources, Inc, Auburn, CA) and lyophilized to ∼5 µl. Peptides were re-suspended in 100 µl strong cation exchange (SCX) buffer A (30% acetonitrile, 5 mM KH_2_PO_4_, pH 2.7). SCX was performed on the entire sample using an Agilent 1100 series HPLC with Chem Station for LC (Agilent Technologies, Santa Clara, CA) using a PolySULFOETHYL column, 2.1 mmID×100 mm, 5 µ, 300 Å (The Nest Group, Southborough, MA). SCX buffer B consisted of buffer A with 350 mM KCL. The gradient was held at 0% B for 9 min and then went from 0% B to 29% B over 54 min, 29% B to 75% B over 45 min, and 75% B to 100% B over 9 min, with a flow rate of 100 µl/min. Fractions were collected every three minutes. The first 5 fractions were pooled, giving a total of ∼50 fractions per sample. Fractions were lyophilized and re-suspended in reverse phase (RP) buffer A (0.5% acetonitrile 0.1% formic acid in H_2_0). One tenth of each individual SCX fractions was injected on a 5 µl loop from a CTC pal auto-sampler (Leap technologies, Carrboro, NC) and peptides loaded onto Vydac Everest C18 column, 300 Å, 5 µm, 100 mm, 500 µm ID (The Nest Group) at 20 µl/min in 100% RP buffer A with an Express C100 system (Eksigent Technologies, Dublin, CA). The mobile phase was diverted to waste for the first 10 min to remove KCl and other salts. The flow rate was then reduced to 12 µl/min and peptides were eluted over a 50 min gradient from 0% to 40% RP buffer B (5% H_2_0, .1% formic acid in acetonitrile). After elution the column was washed for 10 min with 100% RP buffer B and equilibrated for 10 min with 100% RP buffer A. The LTQ mass spectrometer (ThermoFisher Scientific, San Jose, CA) was operated in the positive ion mode using electrospray ionization with a capillary temperature of 200°C. Nitrogen was used as a sheath gas at 41 and an auxiliary gas at 12 (arbitrary units). MS spectra were acquired for a mass range of 400–1200 *m/z* over 30 milliseconds. The top 5 most intense ions per spectra were selected and sequenced with a collision induced dissociation energy (CID) of 35.0 and an activation energy of 25 (arbitrary units). A dynamic exclusion window was applied to prevent the selected ions from being sequenced for 200 ms after the initial acquisition.

### Analysis of LC-MS/MS Data

The data sets were searched against the International Protein Index (IPI) human protein sequence database (version 3.43, number of protein sequences: 72,346, total length of database entries: 30,410,250 amino acid residues) for peptide sequences using SEQUEST 3.1 (ThermoFisher, San Jose, CA). Raw mass spectra were converted to DTA peak lists using BioWorks Browser 3.2 (ThermoFinnigan) with the following parameter settings: peptide mass range 300–5000 Da, threshold 10, precursor mass ±1.4 Da, group scan 1, minimum group count 1, minimum ion count 15. SEQUEST searches specified that peptides should have a maximum of two internal tryptic cleavage sites and possess two tryptic termini, with methionine oxidation and cysteine carbamidomethylation as possible modifications and used a peptide mass tolerance of ±1.4 Da and a fragment ion tolerance of 0. The search results were converted into pepXML format. Peptide identification probabilities were calculated by executing PeptideProphet as implemented in the Trans-Proteomics Pipeline version 2.8 (Institute for Systems Biology, Seattle, WA) [47]. SEQUEST results were processed using the “-Ol” tag, which uses ΔCn* values unchanged.

Results from both biological replicates were combined in a single statistical analysis of protein expression using the EBP 1.0 as described previously [38]. Briefly, EBP estimates both sensitivity and false identification rate and has been validated empirically for LTQ ion trap data using a reversed/forward sequence database search approach [38]. EBP combines the probabilities of correct peptide identification across multiple peptide searches using a function that returns the maximum probability from consensus identifications, and penalizes non-consensual identifications. Replicates are integrated by simultaneously estimating multiple sets of model parameters. Peptides whose sequence matches multiple proteins are integrated in the analysis using “Occam's Razor”, a principle by which the smallest set of probable proteins that is sufficient to explain the peptide sequence identifications is chosen. When proteins cannot be reliably distinguished by unique peptides, they are reported as a protein group. Only proteins with expression probabilities corresponding to a false identification rate of less than 0.01 (1%) were reported (1863 proteins). This was equivalent to expression probabilities of p>0.90 in this data set. One hundred thirty two additional proteins with p>0.90 were members of groups of similar proteins, which could not be distinguished based on the peptide evidence. A separate analysis utilizing a forward/reversed database yielded near identical protein identifications at a 1% false positive cut off (data not shown).

### Validation of Known PSD peptides/proteins

MS/MS spectra of at least two peptides each from 63 PSD proteins (Table 1) were validated by comparison with product ions estimated from ProteinProspector (v4.27.2, http://169.230.19.26:8080/). Peptides from select proteins (Table 1) were subjected to targeted MS/MS analysis to further confirm peptide and protein identifications. These addition spectra were not included in LC-MS/MS data analysis statistics.

### Postmortem tissue collection consent and IRB

Consent for tissue collection was granted prospectively by patients and autopsy consent granted by the next-of-kin at time of death. The postmortem enrollment was conducted under Conte Core A, IRB number 703835 and postmortem protocol was conducted under IRB number 188200 of the University of Pennsylvania.

## Supporting Information

Figure S1 PSD yield is not affected by post mortem interval (PMI), age or freezing time of the brain tissues. Yields of PSD fractions isolated from 12 subjects free of any neuropsychiatric illnesses were plotted against using Method 2. (A): Post mortem Intervals (PMI) (B): Age and (C): Freezer time duration.
(0.81 MB TIF)

Figure S2 Protein-Protein Interactions in PSD fractions are not affected by post mortem interval (PMI), age or freezing time of the brain tissues. Ratios of PSD-95/NR1 signals obtained from the NMDAR1 immunoprecipitation of PSD extracts (fractionated by Method 2) of 12 normal subjects free from neuropsychiatric illnesses were plotted against: (A): Post mortem Intervals (PMI) (B): Age and (C): Freezing time duration.
(0.81 MB TIF)
