# Expression of Concern: The Post-Synaptic Density of Human Postmortem Brain Tissues: An Experimental Study Paradigm for Neuropsychiatric Illnesses

**DOI:** 10.1371/journal.pone.0274661

**Published:** 2022-09-09

**Authors:** The PLOS ONE Editors

Following the publication of this article [1], concerns were raised regarding the results presented in Fig 2C. Specifically, when levels are adjusted to visualize background, there appear to be vertical irregularities suggestive of splice lines in multiple panels presented in this figure.

The corresponding author stated that the blots presented in Fig 2C have not been spliced and noted that in Western blots background noise can be varying in each lane and between each lane, and that demarcations between lines can be the result of exposure artefacts.

The corresponding author stated that the original, uncropped data underlying the Western blots presented their article are no longer available. The electron microscopy images obtained during this study are still available upon request, as are the processed LC-MS/MS data as mzXML files from PeptideAtlas which have been reprocessed in Proteome Discover 2.5, but the raw LC-MS/MS data used in this study are no longer available.

The *PLOS ONE* Editors issue this Expression of Concern to notify readers of the concerns with Fig 2C, which cannot be resolved in the absence of the original Western blot image data.
