# Supplementary figures and images for: The Post-Synaptic Density of Human Postmortem Brain Tissues: An Experimental Study Paradigm for Neuropsychiatric Illnesses
Source: PLoS One. 2009 Apr 16;4(4):e5251. doi: 10.1371/journal.pone.0005251 (PMC2666803; doi:10.1371/journal.pone.0005251)

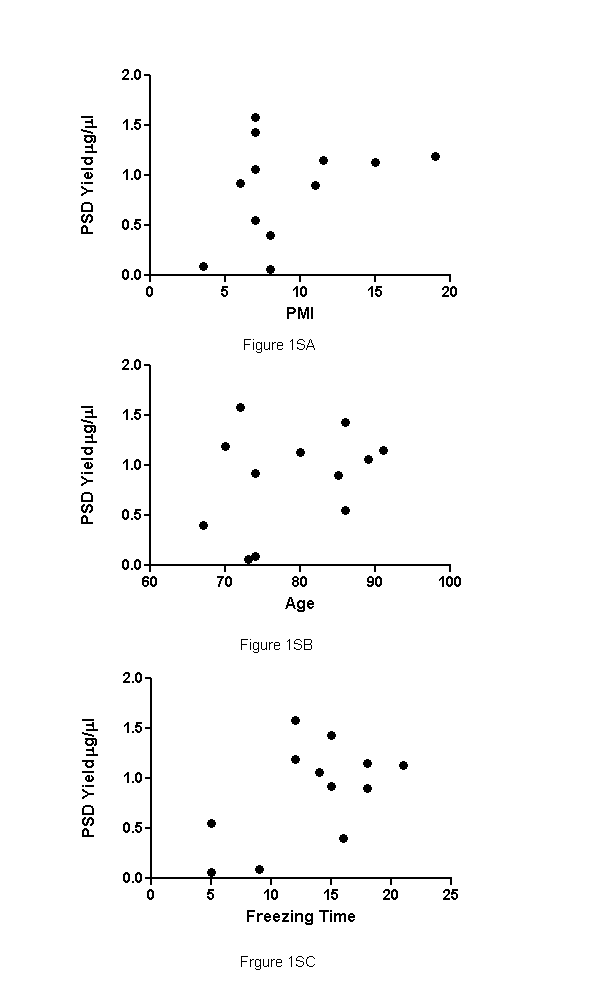

Supplement: Figure S1 — PSD yield is not affected by post mortem interval (PMI), age or freezing time of the brain tissues. Yields of PSD fractions isolated from 12 subjects free of any neuropsychiatric illnesses were plotted against using Method 2. (A): Post mortem Intervals (PMI) (B): Age and (C): Freezer time duration. (0.81 MB TIF) [file pone.0005251.s001.tif]

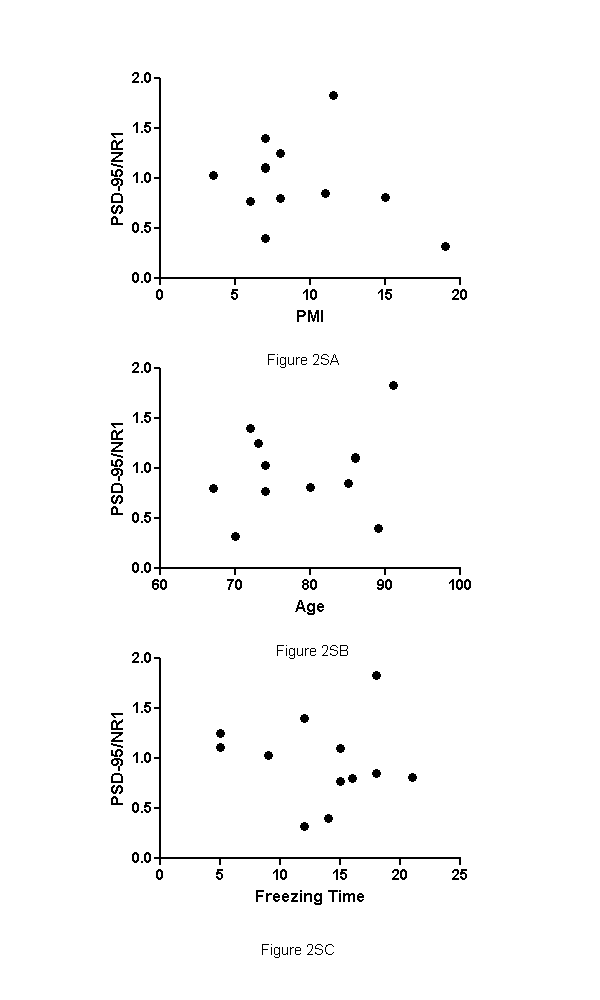

Supplement: Figure S2 — Protein-Protein Interactions in PSD fractions are not affected by post mortem interval (PMI), age or freezing time of the brain tissues. Ratios of PSD-95/NR1 signals obtained from the NMDAR1 immunoprecipitation of PSD extracts (fractionated by Method 2) of 12 normal subjects free from neuropsychiatric illnesses were plotted against: (A): Post mortem Intervals (PMI) (B): Age and (C): Freezing time duration. (0.81 MB TIF) [file pone.0005251.s002.tif]
